# Somatosensory Amplification Scale—Chinese version: psychometric properties and its mediating role in the relationship between alexithymia and somatization

**DOI:** 10.3389/fpsyg.2024.1392351

**Published:** 2024-07-19

**Authors:** Yafei Tan, Xiaoran An, Menglu Cao, Omer Van den Bergh

**Affiliations:** ^1^School of Psychology, Central China Normal University, Wuhan, China; ^2^Key Laboratory of Adolescent Cyberpsychology and Behavior (CCNU), Ministry of Education, Wuhan, China; ^3^Key Laboratory of Human Development and Mental Health of Hubei Province, Wuhan, China; ^4^Faculty of Psychology, Southwest University, Chongqing, China; ^5^Center of Students’ Mental Health, Sichuan Technology and Business University, Chengdu, China; ^6^Health Psychology, University of Leuven, Leuven, Belgium

**Keywords:** Somatosensory Amplification Scale, psychometric properties, somatization, alexithymia, Chinese version

## Abstract

The Somatosensory Amplification Scale (SSAS) was designed to measure individual’s tendency to experience visceral and somatic sensations as unusually intense, disturbing and alarming. In this study, we aimed to investigate the reliability and validity of the SSAS in the Chinese general population, as well as the mediating effect of somatosensory amplification in the relationship between alexithymia and somatization. A total of 386 healthy adults were enrolled in this study. Participants completed the Chinese versions of the Somatosensory Amplification Scale (SSAS-C), the somatization subscale of the Symptom Check List 90 (SCL-90 som), the Toronto Alexithymia Scale (TAS-20), and the Short form Health Anxiety Inventory (SHAI). One hundred and thirty-three participants were randomly selected to complete the SSAS-C again two weeks after the initial assessment. The reliability and validity of the SSAS-C were analyzed. Confirmatory factor analysis showed that the one-factor model achieved adequate model fits; one item was deleted due to low factor loading. The revised SSAS-C showed good internal consistency and test-retest reliability. The SSAS-C scores correlated positively with the scores of SCL-90 som, TAS-20 and the SHAI, showing good convergent validity. In addition, somatosensory amplification mediated the association between alexithymia and somatization. The Chinese version of SSAS has acceptable reliability and validity for the general population. In addition, alexithymia may increase somatization through higher somatosensory amplification.

## Introduction

The term “somatosensory amplification” refers to the tendency to experience normal unpleasant bodily sensations as abnormally intense, noxious, and disturbing (Barsky and Klerman, 1983). It involves three elements: (1) hypervigilance toward bodily sensations; (2) a tendency to focus on certain weak and infrequent somatic and visceral sensations; and (3) a tendency to appraise bodily sensations as more alarming and ominous than they actually are (Barsky et al., 1988). The concept of somatosensory amplification was first proposed as one of the risk factors to explain somatization, health anxiety, and the development of hypochondriasis (Barsky et al., 1988, 1990; Barsky, 1992). Somatization is a tendency to experience somatic distress and symptoms unexplained by organ pathology and to seek medical help for it (Lipowski, 1988; Wise and Mann, 1994; North, 2002). When the somatizing patients assume that they have medical problems, they may focus more on somatic sensations that can confirm their hypothesis (Pennebaker, 1982). Additionally, they may indeed experience more bodily sensations because of anxiety related to their concern on symptoms (Barsky and Wyshak, 1990). The symptoms of patients with somatization are far from minor or trivial, but may result in substantial distress and increased medical costs (Barsky et al., 2005).

To measure somatosensory amplification, the Somatosensory Amplification Scale (SSAS), a ten-item self-report scale, was developed (Barsky et al., 1990). Barsky et al. (1990) hypothesized that somatizing patients are overly disturbed by normal bodily sensations which are not necessarily pathological. Therefore, the scale assesses participants’ sensitivity to mildly uncomfortable bodily experience that is non-pathogenic. It showed good test-retest reliability and internal consistency and close associations with hypochondriasis and somatization tendencies in both healthy and clinical populations (Barsky et al., 1990; Barsky and Wyshak, 1990; Speckens et al., 1996). For example, Takayanagi and Fujiu (2008) suggested an attention bias toward illness in individuals with high SSAS score. Furthermore, a number of studies have reported significant correlations between the SSAS score and self-reported bodily symptoms in psychiatric and psychosomatic outpatients (Spinhoven and van der Does, 1997; Muramatsu et al., 2002; Nakao et al., 2002; Nakao and Takeuchi, 2018; Barsky and Silbersweig, 2023). Based on SSAS, these studies demonstrated a role of somatosensory amplification in explaining why the relation between symptom report and real physiological dysfunction is highly variable (Barsky and Wyshak, 1990).

However, some researchers argued that SSAS was an index of negative reporting style and negative affectivity rather than a specific measurement of somatic sensitivity (Aronson et al., 2001, 2006). For example, it has been found that the SSAS score were highly related to depression and anxiety (Kosturek et al., 1998; Sayar et al., 2005). The SSAS score was also positively linked with alexithymia (Wise and Mann, 1994; Kosturek et al., 1998; Nakao et al., 2002), a personality trait characterized by difficulties in identifying feelings and distinguishing feelings from the physical sensations that accompany an emotional state (Taylor et al., 1991, 1997; Samur et al., 2013; Timoney and Holder, 2013). These arguments can be explained by a theory proposed by Van den Bergh et al. (2017), which suggests that unexplained symptom reports result from the mismatch between the prior prediction in the brain and the actual physiological input. Negative affect and attention bias to bodily signals may increase the gain of the prior. Then relatively weak bodily stimulation may be represented as stronger and more disturbing in individuals with high symptom reports.

The original English version of SSAS has been revised into several languages including French, Farsi, Spanish, Korean, Japanese, Kannada language, Italian, Turkish, and Hungarian (Jimenez Moron and Saiz Ruiz, 1996; Won and Shin, 1998; Nakao et al., 2001b; Duddu et al., 2003; De Berardis et al., 2007; Güleç and Sayar, 2007; Köteles et al., 2009; Bridou and Aguerre, 2013; Aghayousefi et al., 2015). However, there is no validated Chinese form of SSAS up to date. The present study therefore aimed to introduce the SSAS into China and examine the reliability and validity of the SSAS in the Chinese context. In the original version, the researchers proposed that somatosensory amplification is a unitary structure with some degree of stability over time. However, the French revision proposes a two-factor model, assuming that the SSAS items boil down to two factors: Exteroceptive sensitivity and Interoceptive sensitivity (Bridou and Aguerre, 2013). So, we also compared the fit of the two models to determine whether somatosensory amplification is a unitary structure representing somatosensory amplification.

In addition, a number of studies have demonstrated a close relationship between alexithymia and somatization in clinical and community samples (Cox et al., 1995; Deary et al., 1997; Bailey and Henry, 2007; Tam and Wong, 2013; Lanzara et al., 2020). For example, high-level somatization patients had significantly higher alexithymia scores than those with low levels of somatization (Joukamaa et al., 1995; Lanzara et al., 2020). Similarly, in a quantitative review of related articles, it was found that the total alexithymia score was positively correlated with different physical symptom reporting indicators, and individuals suffering from somatoform conditions were significantly more alexithymic than healthy controls, with effect sizes ranging from moderate to large (De Gucht and Heiser, 2003). Although both individuals with alexithymia and somatizing individuals have a tendency to frame psychological discomfort in physical terms (Lanzara et al., 2020), the underlying mechanism of how alexithymia may lead to somatization is still unclear. Psychopathology such as depression (Allen et al., 2011) and distress symptoms (Lanzara et al., 2020) have been proposed as mediators, but it could only partially explain the relationship between alexithymia and somatization. Some scholars have pointed out that alexithymia is a continuous and significant indicator that can predict somatization (Bach and Bach, 1995). An individual with normal emotional processing functions will be able to identify bodily sensations as part of the arousal accompanying emotions and qualify these sensations as secondary (Tyrer, 1973; Barsky and Klerman, 1983). However, alexithymic individuals fail to use emotions as signals in information processing, and instead may pay more attention to the normal physiological arousal accompanying emotions and regard them as primary phenomena and as alarming and ominous (Krystal, 1990). This somatosensory amplification may in turn lead to somatization. As stated, many studies have revealed a positive association between somatosensory amplification and alexithymia (Wise and Mann, 1994; Kosturek et al., 1998; Jones et al., 2004). There is also ample evidence showing the positive relationship between somatosensory amplification and somatization (Güleç and Sayar, 2007; Bridou and Aguerre, 2013). Considering individual variations in both alexithymia and somatization have been associated with somatosensory amplification, the latter may act as a mediator in explaining the relationship between alexithymia and somatization.

Overall, targeting general population, the aims of this study were to (1) translate the SSAS into Chinese; (2) investigate the psychometric properties of the Chinese version of the SSAS; (3) further validate the SSAS-C as a measurement for somatosensory amplification through examining the mediating effect of somatosensory amplification in the relationship between alexithymia and somatization.

## Materials and methods

### Participants

Four hundred participants were recruited from the general population via online advertisements. The inclusion criteria were: age between 18 and 65 years, not pregnant, no IQ problem (i.e., able to read and understand a text), no psychotic and physical disorders. Somatosensory is immature in childhood and adolescence (Taylor et al., 2016). The elderly population may suffer sensory decline, which affect process of somatosensory (Heft and Robinson, 2017). To keep homogeneous in the present study, we only include participants aged between 18 and 65 years old. Participants were assessed using the Health History Inventory of the Body Perception Questionnaire, which asked participants to report the extent to which they are experiencing, have experienced, or have been diagnosed with psychotic and physical symptoms, such as migraine headaches, back problems, asthma, clinical depression and gastric distress or digestive problems (Porges, 1993). Participants were excluded if they reported “Usually” or “Always.” Excluding incomplete responses, a final sample of 386 subjects (160 males; age = 31.59 ± 11.92 years) were used for analysis. Of these, 133 participants (55 males; age = 30.08 ± 11.84 years) were available for follow-up and completed the Chinese version of the SSAS again two weeks after the initial assessment. The study’s protocol was approved by the Ethical Committee of Central China Normal University (IRB Number: CCNU-IRB-202204001). All participants provided informed consent and disclosed no conflicts of interests. The procedures in this study were performed in accordance with the Declaration of Helsinki.

### Measures

#### Chinese version of the Somatosensory Amplification Scale (SSAS-C)

The SSAS is a 10-item self-report scale developed by Barsky et al. (1990). Respondents are asked to score each item on a scale from 1 (*Not at all true*) to 5 (*Extremely true*) according to how well each item describes themselves in general. A total amplification score is obtained by adding up the scores for each item. A higher total score indicates a greater tendency to amplify somatic sensations.

After obtaining the permission from the developer of the original English version of the SSAS, the Chinese version of the SSAS was obtained through to the standard back-translation procedures (Brislin, 1970). First, the scale was translated into Chinese by two independent bilingual authors. This preliminary version was then evaluated by three psychologists. They verified the clarity of the Chinese items and evaluated whether relevant cultural adaptations were needed, resulting in a combined forward-translation. It was then translated back into English by a professional translator. The original developer of the SSAS checked this back-translated version and confirmed that the meaning of the original version had not been changed or lost. Consequently, the final version of the SSAS in Chinese (SSAS-C) was established and used in the present study.

#### Somatization subscale of the Symptom Check List 90 (SCL-90 som)

This study used the Chinese somatization subscale of the Symptom Check List 90 (SCL-90 som) to measure somatization propensity (Derogatis et al., 1973; Wang, 1984). The somatization subscale consists of twelve items assessing the intensity of symptoms experienced during the previous week (e.g., nausea or upset stomach). Each item is rated on a scale ranging from 0 (*not at all distressed by the item*) to 4 (*extremely distressed by the item*). The total score was calculated across all 12 items, with a higher score reflecting a greater somatization tendency. The Chinese version of the SCL-90 som used in the current study showed a Cronbach’s α of 0.89.

#### Toronto Alexithymia Scale (TAS-20)

The Chinese version of the 20-item Toronto Alexithymia Scale (TAS-20) developed by Yi et al. (2003) was used. The TAS-20 evaluates the individual’s lack of emotional awareness (Bagby et al., 1994), consisting of three subscales: Difficulty Identifying Feelings (TAS-1), Difficulty Describing Feelings (TAS-2) and Externally-Oriented Thinking (TAS-3). The TAS-1 includes 7 items assessing individual’s ability to identify their feelings and to distinguish the feelings from the bodily sensations accompanying emotions (e.g., “I have feelings that I can’t quite identify”). The TAS-2 comprises 5 items measuring the ability to describe feelings (e.g., “It is difficult for me to reveal my innermost feelings, even to close friends”). The TAS-3 consists of 8 items evaluating individual’s tendency to externally oriented thinking (e.g., “I prefer talking to people about their daily activities rather than their feelings”). Participants answer each item on a Likert-type scale ranging from 1 (*strongly disagree*) to 5 (*strongly agree*). The Chinese version of the TAS-20 has shown good validity and reliability in several studies (e.g., Yang et al., 2008, 2009). The internal consistency was good in the current study, with a Cronbach’s α of 0.82. The Cronbach’s α of the three subscales were 0.86, 0.65 and 0.23, respectively. Consistent with previous studies, the Cronbach’s α of TAS-3 was also lower than the other two subscales (Yi et al., 2003).

#### Short Health Anxiety Inventory (SHAI)

Health anxiety was measured using the Chinese version of the Short Health Anxiety Inventory (Zhang et al., 2013). The SHAI is a self-report inventory that contains 18 items assessing health-related anxiety not due to physical health status (Salkovskis et al., 2002). Each item contains four different statements and participants are asked to select the one which best describes their feelings over the past six months. The score for each item is ranging from 0 to 3, resulting in a maximum total score of 54. The SHAI showed a Cronbach’s α of 0.89 in the current sample.

### Data analyses

The data were analyzed using the Statistical Product and Service Solutions version 27.0 (SPSS 27.0) and IBM SPSS Amos 26. We conducted a confirmatory factor analysis (CFA) to examine the factor structure of Chinese version of the SSAS. The following goodness-of-fit indices were used to evaluate model fit: relative chi-square (X ^2^/df), root mean square error of approximation (RMSEA), comparative fit index (CFI), Tucker-Lewis index (TLI), and standardized root mean square residual (SRMR) (Hu and Bentler, 1999). A range from 5.0 to 2.0 of X ^2^/df ratio is acceptable (Hooper et al., 2008). Values for TLI and CFI of greater than 0.90 are acceptable (Sharma et al., 2005; Hair et al., 2010). It was thought that a score for RMSEA of below 0.08 shows a good fit (MacCallum et al., 1996). A value for SRMR of below 0.05 indicate fair fit (Byrne, 1998; Diamantopoulos and Siguaw, 2000). Larger value of CFI and TLI, and smaller value of RMSEA and SRMR indicate that the model fits better. One-way ANOVA were used to make sociodemographic comparisons and item discrimination. To examine the reliability of SSAS-C, the internal consistency was assessed using item-total correlations and Cronbach’s alpha coefficient. Moreover, we also reported McDonald’s ω (McDonald, 1999) for the SSAS-C, which has been suggested as a better indicator of reliability (Teo and Fan, 2013). A ω value of greater than 0.70 reflects good reliability (Field, 2013). In addition, the test-retest consistency was assessed using intra-class correlations (ICC), with values below 0.50 signifying poor reliability, 0.50 to 0.75 representing moderate reliability, above 0.75 indicating good reliability (Koo and Li, 2016). The concurrent validity of the SSAS-C was examined by calculating Pearson correlations between the SSAS-C score and the scores of SCL-90 som, TAS-20 and SHAI, respectively. To examine the mediating role of somatosensory amplification in the relationship between alexithymia and somatization, we used PROCESS v4.0 prepared by Hayes in SPSS 27.0. To this end, alexithymia served as a predictor and the somatization served as the outcome. The bootstrapping method based on 5,000 bootstrap resamples was used to assess 95% bias-corrected confidence intervals of indirect effects for the mediation model. Finally, the alpha-level of significance for all statistical analyses was set to 0.05 in this study.

## Results

### Confirmatory factor analysis

**FIGURE 1:**
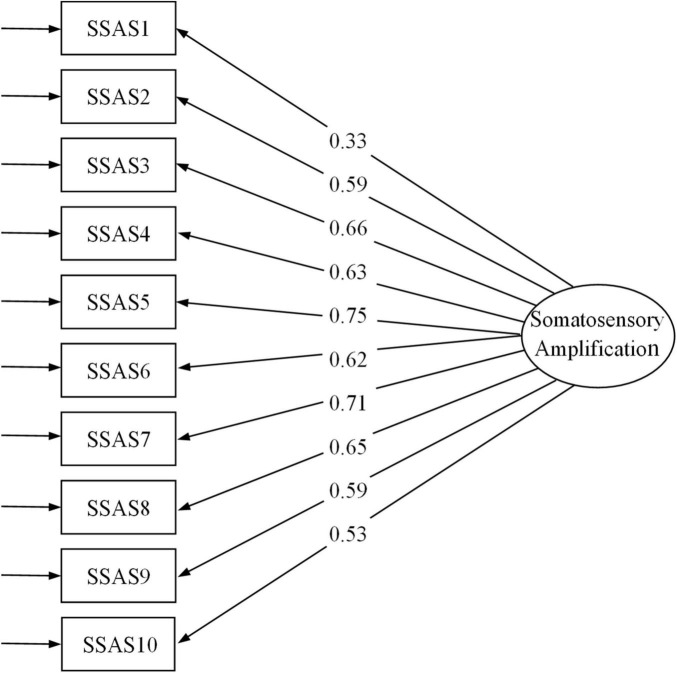
Factor loading of the Somatosensory Amplification Scale. SSAS, Somatosensory Amplification Scale.

There are two viewpoints about the structure of SSAS, respectively, the one-factor model in the original version and the two-factors model in the French revised version. The one-factor model considers that SSAS items are represented by one global dimension called somatosensory amplification. The two-factors model considers that SSAS items are represented by two factors: Exteroceptive sensitivity (items: 2, 4, 5, 7, 8, 9, 10) and Interoceptive sensitivity (items: 1, 3, 6). In this study, the results of CFA for the two models were compared and all of the fit indices for the two models are shown in Table 1. It shows that both models for the SSAS fit the sample data well. As shown in Figure 1, the factor loading of item-1 (*When someone else coughs, it makes me cough too*) was 0.33, which explains 10% latent variable variance (*R*^2^ = 0.1). Chin (1998) describes R^2^ value of 19% or below as weak. Therefore, the item-1 was deleted for further analysis. The results of CFA for the 9-item SSAS-C show improved model fit compared to the 10-item SSAS-C (Table 1). However, the estimated correlation between factor 1 and factor 2 of the two-factor model for both 10-items and 9-items SSAS-C was 1.03, indicating the two factors were not statistically distinguishable (Handscomb et al., 2016). More importantly, the one-factor model was more concise and consistent with the original English version (Barsky et al., 1990). Therefore, the one-factor model is more appropriate than the two-factor model.

**TABLE 1:** Goodness of fit measures for the two compared models of the Somatosensory Amplification Scale—Chinese version (*N* = 386).

		X^2^/df	RMSEA	CFI	TLI	SRMR
10 items	1-factor model	3.323	0.078	0.931	0.912	0.0479
	2-factor model	3.408	0.079	0.931	0.908	0.0479
9 items	1-factor model	3.096	0.074	0.949	0.933	0.0438
	2-factor model	3.202	0.076	0.949	0.929	0.0438

RMSEA, root mean square error of approximation; CFI, comparative fit index; TLI, Tucker-Lewis index. SRMR, standardized root mean square residual.

### Item discrimination

To analyze the item discrimination of the revised Chinese version of the SSAS, we quartered the group according to the total score of the scale and compared the four quartiles: 0–25%, 25–50%, 50–75%, 75–100% using one-way ANOVA and Least Significance Difference (LSD). The results showed that each item had a good discrimination among the four groups (Table 2).

**TABLE 2:** Item discrimination.

	Group M (SD)				*F*	*p*-value	LSD
	0∼25% (*n* = 97)	25∼50% (*n* = 96)	50∼75% (*n* = 96)	75∼100% (*n* = 97)			
Item 1	1.45 (0.90)	1.97 (0.95)	2.01 (0.92)	2.34 (1.10)	13.74	< 0.001	1 < 2 = 3 = 4
Item 2	2.24 (1.24)	3.35 (1.10)	3.77 (0.91)	4.28 (0.76)	69.84	< 0.001	1 < 2 < 3 < 4
Item 3	2.08 (0.99)	3.00 (0.90)	3.47 (0.69)	3.81 (0.68)	79.09	< 0.001	1 < 2 < 3 < 4
Item 4	1.87 (0.97)	2.79 (0.97)	3.33 (0.99)	3.97 (0.82)	86.54	< 0.001	1 < 2 < 3 < 4
Item 5	2.41 (1.25)	3.73 (0.78)	4.08 (0.47)	4.48 (0.50)	116.62	< 0.001	1 < 2 < 3 < 4
Item 6	2.03 (1.12)	2.95 (0.91)	3.40 (0.88)	3.87 (0.86)	65.44	< 0.001	1 < 2 < 3 < 4
Item 7	2.46 (1.24)	3.68 (0.73)	4.07 (0.63)	4.38 (0.56)	97.50	< 0.001	1 < 2 < 3 < 4
Item 8	1.82 (0.99)	3.02 (0.88)	3.43 (0.96)	3.87 (0.81)	89.42	< 0.001	1 < 2 < 3 < 4
Item 9	1.79 (0.95)	2.68 (1.00)	3.05 (0.92)	3.87 (0.88)	80.83	< 0.001	1 < 2 < 3 < 4
Item 10	1.81 (0.99)	2.66 (0.96)	2.91 (1.00)	3.68 (1.04)	57.09	< 0.001	1 < 2 = 3 < 4

Group 1: 0∼25%, group 2: 25∼50%, group 3: 50∼75%, group 4: 75∼100%; LSD, Least Significance Difference.

### Descriptive analysis

The results indicate significantly higher 9-item SSAS-C scores at retest (M = 32.60, SD = 4.31) compared to the scores at the first time (M = 29.45, SD = 5.59; *t* = −7.93, *p* < 0.001). Table 3 shows the demographic characteristics of the participants and the scores on the revised 9-item SSAS-C in different groups for both the test sample at the first time (Time 1) and the retest sample (Time 2). Women reported significantly higher scores on SSAS-C than men in this study [*t*(384) = −3.35, *p* = 0.01]. In the subgroups of age and education, we found that individuals in the age range of 18 to 30 years had the highest SSAS-C scores and individuals who had a higher level of education reported higher SSAS-C scores.

**TABLE 3:** Characteristics of the participants and the Chinese version of Somatosensory Amplification Scale scores.

Characteristic	*N* (%)		SSAS-C score M (SD)		*p*-value	
	Time 1	Time 2	Time 1	Time 2	Time 1	Time 2
Gender					< 0.001	= 0.049
Male	160 (41.45%)	55 (41.35%)	27.04 (7.89)	31.73 (4.75)		
Female	226 (58.55%)	78 (58.65%)	29.57 (6.47)	33.22 (3.88)		
Age (SD)					= 0.019	= 0.218
18–30 years	233 (60.36%)	92 (69.17%)	29.33 (6.73)	33.03 (4.06)		
30–50 years	126 (32.64%)	29 (21.80%)	27.10 (8.02)	31.52 (5.07)		
50–65 years	27 (7.00%)	12 (9.03%)	28.11 (6.07)	31.92 (3.94)		
Education					= 0.002	= 0.021
Middle school and below	46 (11.92%)	15 (11.28%)	25.19 (8.27)	29.73 (3.49)		
High school	59 (15.28%)	14 (10.53%)	28.10 (6.45)	32.57 (5.77)		
University and above	281 (72.80%)	104 (78.19%)	29.14 (7.02)	33.02 (4.07)		

SSAS-C, Chinese version of Somatosensory Amplification Scale.

### Reliability

The item–total correlations are shown in Table 4. It should be noted that the item-1 had a factor loading of less than 0.5 and the Cronbach’s alpha of the entire scale increased after it is removed. This result suggested to delete item-1; all the following results are for the 9-item SSAS-C. The average inter-item correlation for 9 items was 0.40, with a range of 0.25–0.53. The Cronbach’s alpha of the 9-item SSAS-C score was 0.86. The ω values for the 9-item SSAS-C was 0.85. These values indicate good reliability of the 9-item SSAS-C. The 2-week test-retest reliability indexed by ICC for the SSAS-C was 0.65, indicating moderate test-retest reliability.

**TABLE 4:** Somatosensory Amplification Scale internal consistency evaluation, the impact of each item on the scale and Cronbach’s α values if item deleted.

SSAS item	Corrected item–total correlation	α if item deleted
1. When someone else coughs, it makes me cough too	0.45***	0.86
2. I can’t stand smoke, smog, or pollutants in the air	0.65***	0.84
3. I am often aware of various things happening within my body	0.68***	0.84
4. When I bruise myself, it stays noticeable for a long time	0.69***	0.84
5. Sudden loud noises really bother me	0.76***	0.83
6. I can sometimes hear my pulse or my heartbeat throbbing in my ear	0.66***	0.84
7. I hate to be too hot or too cold	0.72***	0.83
8. I am quick to sense the hunger contractions in my stomach	0.69***	0.84
9. Even something minor, like an insect bite or a splinter, really bothers me	0.66***	0.84
10. I have a low tolerance for pain	0.61***	0.85

***p < 0.001. SSAS, Somatosensory Amplification Scale.

### Concurrent validity

Correlations between the total scores of the SSAS-C and scores for other measurements (SCL-90 som, TAS-20, and SHAI) are shown in Table 5. As expected, the total scores of the SCL-90 som, the TAS-20 and the SHAI correlated positively with the total scores of the 9-item SSAS-C. However, the associations of SSAS-C scores with SCL-90 som (*r* = 0.29, *p* < 0.001) and SHAI scores (*r* = 0.28, *p* < 0.001) are moderate (Cohen, 1992).

**TABLE 5:** The correlations between the Somatosensory Amplification Scale and other measurements (*N* = 386).

	SCL-90 som	TAS-20	TAS-1	TAS-2	TAS-3	SHAI
SSAS	0.29***	0.37***	0.50***	0.28***	−0.01	0.28***
Mean (SD)	6.17 (5.73)	54.16 (9.63)	18.20 (5.32)	13.98 (3.31)	21.98 (3.18)	14.14 (7.54)

***p < 0.001. SSAS, Somatosensory Amplification Scale; SCL-90 som, Symptom Check List somatization subscale; TAS-20, Toronto Alexithymia Scale; TAS-1, Difficulty Identifying Feelings; TAS-2, Difficulty Describing Feelings; TAS-3, Externally-Oriented Thinking; SHAI, Short Health Anxiety Inventory.

### Mediation analysis

**FIGURE 2:**
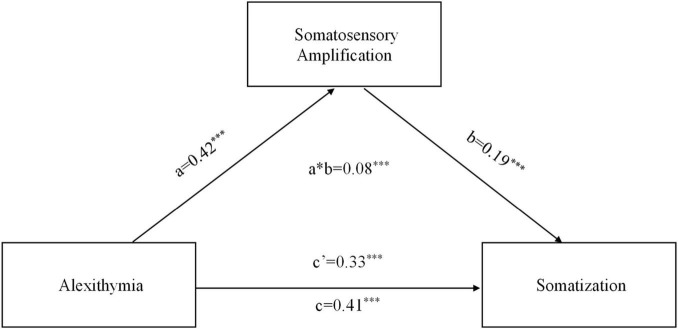
Results of Mediation analysis. a, initial variable; b, outcome; c, simple a to b effect when we do not control for the mediator; c’, x to y effect when we do control for the mediator; a*b, mediation effect (indirect). The path weights in the graph were standardized and obtained after controlling for gender, age, and education level. ***p < 0.001.

As shown in Figure 2, after controlling for demographic covariates (i.e., gender, age, and education level), alexithymia significantly and positively predicted somatosensory amplification (*p* < 0.001), which in turn significantly and positively predicted somatization (*p* < 0.001). The positive direct association between alexithymia and somatization remained significant, showing that somatosensory amplification partially mediated the relationship between alexithymia and somatization. The indirect effect was 0.08 (95% CI: 0.02, 0.07). In addition, somatosensory amplification also partially mediated the associations of somatization with Difficulty Identifying Feelings (indirect effects = 0.06, 95% CI = 0.01–0.11) and Difficulty Describing Feelings (indirect effects = 0.08, 95% CI = 0.08–0.22), respectively.

## Discussion

Somatosensory amplification is a major explanatory construct for various forms of somatoform disorders. The present study validated the Chinese version of the Somatosensory Amplification Scale (SSAS-C). The findings suggested that a slightly revised version of the SSAS (deleting its first item) had sound psychometric properties such as good reliability and convergent validity in a Chinese non-clinical sample. Furthermore, the scale showed a gender difference, in which women had higher levels of somatosensory amplification. Finally, the level of somatosensory amplification measured by SSAS-C showed a mediating effect on the relationship between alexithymia and somatization.

Barsky and Klerman (1983) defined somatosensory amplification as “a tendency to experience somatic and visceral sensation as unusually intense, noxious, and disturbing.” It has been conceptualized as a unidimensional construct and assessed by the SSAS with a one-factor model (Barsky et al., 1990). Consistent with the results reported in most previous studies (Won and Shin, 1998; Nakao et al., 2001b; Duddu et al., 2003; De Berardis et al., 2007; Güleç and Sayar, 2007; Köteles et al., 2009; Bridou and Aguerre, 2013), the current study showed that the one-factor model fitted the sample data well and was more appropriate than the two-factor model (Bridou and Aguerre, 2013). In addition, the confirmatory factor analysis revealed that the factor loading of item-1 “When someone else coughs, it makes me cough too” was lower than 0.5. Whereas all other items describe the perception of processes related to one’s own body, item-1 deals with the impact of other people’s bodily processes on one’s own behavior. Other previous studies have reported similar results, showing the item-1 had relatively low factor loading (Barsky et al., 1990; Jasper et al., 2013). Another potential explanation is that this phenomenon may reflect empathy or social rules in the Chinese collectivist cultural context rather than individual somatic sensitivity. Therefore, we deleted this item, resulting in a 9-item Chinese version of SSAS. The 9-item SSAS-C showed satisfactory internal consistency (Cronbach’s alpha). It would be interesting to investigate the cultural difference of somatosensory amplification using data from different culture groups in future studies. Test-retest consistency of the 9-item SSAS-C was assessed in 2 weeks indexed by ICC. The results indicate a moderate test-retest reliability, which replicates the finding of Güleç and Sayar (2007). We also found significantly higher SSAS-C scores at retest compared to the scores at the first time. This may be caused by the relatively short interval between two tests in the present study. It is possible that the test of SSAS at the first time induced them to pay more attention to somatosensory signals in anticipation of the second test, which may have increased the ratings for the items in the retest.

Moreover, in the current study, somatosensory amplification assessed by the SSAS-C showed the expected correlations with theoretically related measures. Specifically, as somatosensory amplification is a reliable indicator of somatization (Nakao and Barsky, 2007), a significantly positive correlation between the SSAS-C and the SCL-90-R somatization subscale was revealed. However, this correlation was moderate (Cohen, 1992), which replicates the finding of Bridou and Aguerre (2013). This might be due to the relatively low average and low variability in somatization scores of our sample constituting mainly university students, as well as to the difference in focus between the scales: SSAC is more about beliefs and attitudes about symptoms, whereas SCL-90 som focuses more on the symptoms themselves. Consistent with previous literature (Barsky et al., 1990), the results showed a significantly positive correlation between SSAS-C and SHAI. It suggests that a greater tendency to worry about health tends to be associated with a greater propensity to focus on, and thereby maximize, bodily sensations. On the other way around, cognitive misinterpretations of bodily sensations may cause health anxiety (Marcus et al., 2007). Additionally, we found that SSAS-C score was positively related to the level of individual alexithymia. More specifically, the SSAS was correlated with dimensions of Difficulty Identifying Feelings and Difficulty Describing Feelings, respectively, but not with dimension of Externally-Oriented Thinking. This corresponds to some previous studies indicating that the TAS-20 factor of “externally oriented thinking” was not closely correlated with the TAS-20 total score and other two factors (Fukunishi et al., 1997; Kojima et al., 2001; Leising et al., 2009), nor with the tendency to somatization (De Gucht and Heiser, 2003; Bailey and Henry, 2007; Mattila et al., 2008; Tam and Wong, 2013).

In terms of gender differences, the total score of SSAS was significantly higher in females than in males. Therefore, it would be appropriate to make gender comparisons when using the SSAS-C. This is generally in line with previous research on somatosensory amplification (Bridou and Aguerre, 2013; Nakao and Takeuchi, 2018). This finding further indicates that women are more sensitive to bodily stimuli than men, and therefore may experience more somatic distress and report a greater number of somatic symptoms (Nakao et al., 2001a).

Finally, somatic amplification was found to mediate the association between alexithymia and somatization. These findings were consistent with previous studies showing that the somatization is accompanied by alexithymia (Nakao et al., 2002; Jones et al., 2004; Güleç and Sayar, 2007). Alexithymia refers to difficulties in both identifying and expressing emotions (Nemiah and Sifneos, 1970; Taylor et al., 1997; Samur et al., 2013; Timoney and Holder, 2013). It has been defined as a possible personality risk factor for a variety of medical and psychiatric disorders involving emotional regulation problems (Taylor et al., 1991; Lumley et al., 1996; Porcelli et al., 2013; Luminet et al., 2018). Impaired emotional processing and emotion regulation capacity underlying alexithymia may lead to a focus on, amplification and misunderstanding of somatic sensations that accompany emotional arousal, thus resulting in somatization seeking medical help for medically unexplained symptoms (Pennebaker, 1982; Wise and Mann, 1994; Orrù et al., 2020). It is worthy to note that somatosensory amplification could only partially explain the relationship between alexithymia and somatization. This is consistent with previous studies showing that depression (Allen et al., 2011) and distress symptoms (Lanzara et al., 2020) mediated the alexithymia-somatization association.

### Limitations and future directions

These findings of the present study must be interpreted considering a number of limitations. First, the sample in present study constituted mainly university students, which limits the application of the findings to the general population. In addition, somatic amplification was introduced as a clinical construct in relation to somatization and hypochondriasis, but whether the SSAS-C is applicable to Chinese clinical samples is still unknown. Future studies are recommended to validate the SSAS in more representative Chinese population as well as clinical sample. Second, the retrospective nature of this study design makes it difficult to draw clear conclusion on the direction of the relationship between alexithymia, somatization, and somatic amplification. Longitudinal study design is needed to disentangle the associations between somatization, alexithymia, and variations in somatic amplification. Finally, the data collected in this research is self-report. In the future, its validity as a measure of sensitivity to non-pathological bodily sensation should be established by comparing it with direct, objective measures of somatic and visceral perception.

## Conclusion

The current study provides evidence for good psychometric properties of the SSAS-C in a Chinese non-clinical sample. In addition, somatosensory amplification measured by the SSAS-C was found to mediate the association between alexithymia and somatization. The SSAS is a potential tool for further investigation in the field of psychiatric disorders that are accompanied by functional somatic complaints. Moreover, the SSAS-C can be helpful in examining the cultural differences in somatosensory amplification and clarifying the relationships among somatosensory amplification, alexithymia and somatization in differing cultural contexts.

## Data availability statement

The raw data supporting the conclusions of this article will be made available by the authors, without undue reservation.

## Ethics statement

The studies involving humans were approved by the Ethical Committee of Central China Normal University (IRB Number: CCNU-IRB-202204001). The studies were conducted in accordance with the local legislation and institutional requirements. The participants provided their written informed consent to participate in this study.

## Author contributions

YT: Conceptualization, Funding acquisition, Investigation, Project administration, Supervision, Writing – original draft. XA: Conceptualization, Data curation, Methodology, Writing – original draft. MC: Conceptualization, Methodology, Supervision, Writing – review & editing. OV: Supervision, Writing – review & editing.
